# Biomechanics of landing in gymnasts: a scoping review

**DOI:** 10.3389/fspor.2025.1602058

**Published:** 2025-06-06

**Authors:** Kateřina Pavlasová, Lucia Bizovská, Aleš Gába, Roman Farana, Miroslav Janura

**Affiliations:** ^1^ Department of Natural Sciences in Kinanthropology, Faculty of Physical Culture, Palacký University Olomouc, Olomouc, Czechia; ^2^ Department of Human Movement Studies, Faculty of Education, University of Ostrava, Ostrava, Czechia

**Keywords:** biomechanics, gymnastics, landing, muscle function, stability

## Abstract

This scoping review aimed to map methodologies used to assess landing biomechanics in gymnasts, focusing on muscle function and stability. Four research questions were formed, addressing common methodological approaches, factors affecting stability, and the relationships between muscle function, strength, and stability during landing. The searches were conducted across six databases and supplemented by reference and forward citation searches. Eight studies met the inclusion criteria, encompassing 212 participants aged 8–25 years, predominantly competitive gymnasts. The studies revealed significant variability in methods for assessing postural stabilization and muscle function during landing. Stabilization was evaluated using time to stabilization and center of pressure metrics, while muscle activity was predominantly measured via surface electromyography, focusing on lower limb muscles. Factors such as drop height, age, training level, and task-specific demands influenced muscle activity patterns but were inconsistently reported. Gymnasts demonstrated superior neuromuscular control compared to untrained individuals, with distinct muscle activation patterns during landing phases. Despite these insights, no studies examined the interplay between muscle strength, activity, and stabilization metrics. The lack of standardized methodologies limits direct comparisons and generalizations. This review highlights the need for consistent protocols and further research to explore relationships between muscle function, stability metrics, and performance outcomes in gymnastics.

## 1 Introduction

A quality landing after performing a jump, acrobatic element, acrobatic series or a dismount is necessary to achieve high scores and ensure the safety of gymnasts (1–3). Errors during the landing are evaluated according to the international rules (4) and include, for example, loss of balance followed by a step of the lower limb, additional arm movements, small foot movements, spreading of the lower limbs, landing in a deep squat or incomplete completion of a turn (5). At the elite level, gymnasts are exposed to intense workloads, training 21–37 h per week (6) with performing around 700–1,300 elements daily (7). Older sources provided estimates of gymnasts performing 200 landings per week (8). Marinšek (3), who examined the landing success rate of male gymnasts at the 2004 European Championships, found that only 30% were performed flawlessly. Most landings are performed on the floor due to routines consisting of many acrobatic and gymnastic elements (9) as opposed to the vault where a maximum of two jumps are performed in a competition (10).

The continuous modernization of the equipment leads to a constant increase in the difficulty and complexity of the gymnastic elements and routines created from them. This in turn translates into increased demands for stabilization of the landing, for which a high degree of postural and neuromuscular control, optimal muscle coactivation and efficient use of muscle strength are important (11). The loading that gymnasts must endure during the landing, based on measurements of the magnitude of the vertical component of the ground reaction force, ranges from 7.1–15.8 times their body weight and varies with height, complexity of the acrobatic element and the execution of the landing (12). This is related to the different strategies that can be implemented during the process of postural stabilization. According to the maximum knee joint angle achieved, the landing strategy can be divided into “soft” and “stiff” (3, 13, 14). The “soft” landing, typical for recreational gymnasts, is characterized by an angle at the knee joints of more than 63°. Higher level gymnasts use “stiff” landings (angle lower than 63°) to reduce loading on the heels (15) leading to the increase of ankle stability (3). Different landing strategies can also be seen depending on sex. Although the rules consistently penalise the aforementioned errors in landing related to upper body movements or step execution, the rules differ for the position of the lower limbs with respect to sex. For women, even a slight spread in the landing position will result in a point deduction, while for men it is allowed without a point loss. Straker et al. (16) in their study also pointed out the higher range of movement in the knee and hip joints in the execution of the men's landing with inter-individual differences in performance. Female athletes tend to perform the landing tasks with a more erect landing posture, and less knee and hip ﬂexion (17).

Only well physically prepared male and female gymnasts are able to perform gymnastic elements technically correctly and with minimal risk of injury (18). Increasing the efficiency of the stabilization of the landing can be achieved by flexion and extension in the joints of the lower limbs and trunk (3, 19). The muscles of the lower limbs and trunk ensure optimal absorption of the applied forces, preventing excessive loading of the musculoskeletal system (20–22). A balanced ratio of muscle strength between agonist and antagonist groups is also important for lower limb joint stability and injury prevention in the event of overload (23). Gymnastic training not only improves muscle functions, it also streamlines the process of postural control (18, 24). However, the association between postural control and muscle function has not been demonstrated in healthy subjects (25). Consistently, the published studies (26–30) point to the fact that postural control is task-specific and a gymnastic proficiency may not imply a general improvement in postural stability in the sense of a reduction in postural titubations. While previous studies have examined stabilization in balance recovery tasks (31), stabilization specifically during landing remains unexplored.

Biomechanical analysis of jumps and landings has been described in several previous works in general, e.g., Ericksen et al. (32) and Prassas et al. (33). Other studies have addressed injury risk and prevention (19, 22, 34–40) or have provided comprehensive summaries and analyses of relevant sources (41). However, none of the above studies have focused on the aspect of stability during landing in relation to the neuromuscular control associated with the process of stabilization during landing.

## 2 Objective

In an effort to bridge the evidence gap, the aim of this scoping review was to collect and map methods used for assessment of the biomechanics of landing in gymnasts, with a key focus on muscle function and stability during landing. Landings after performing jumps or acrobatics elements on the floor were considered for the purpose of this review. Following review questions were formed:

1. What methodological approaches are most often used for assessment of stability during landing in gymnasts?
2. What factors affect the stability during landing in gymnasts?
3. What methodological approaches are most often used for assessment of lower limb and trunk muscles' function (e.g., strength, activity) during landing in gymnasts?
4. Which specific lower limb and trunk muscles' strength or activation levels are most frequently linked to landing stability in gymnasts?

## 3 Methodology

The design and reporting of this scoping review follows the Preferred Reporting Items for Systematic Reviews and Meta-Analyses extension for scoping reviews [PRISMA-ScR, Tricco et al., (42), see Supplementary Table 1 for a checklist]. The protocol was registered with the Open Science Framework on April 18, 2024 and it is accessible at https://osf.io/terb2.

### 3.1 Search strategy

The original search strategy was created by AG, LB, and KP and was designed to identify experimental and observational studies published before March 2024 in the following databases: Cochrane Central, MEDLINE, SPORTDiscus, ProQuest, Scopus, and Web of Science, where KP searched. Detailed search strategy for each database is included in the study protocol (43). The key words are representing four domains this review is focused on—gymnastics, landing, stability, and muscle function. Because of the low coverage of synonyms in the MeSH database, several specific terms that increase the sensitivity of the search were used. The ResearchRabbit application (Human Intelligence Technologies Inc., Seattle, WA, USA) was used by LB for a forward reference search, employing all eligible studies from the database search as seeding articles. All reference lists of included studies were reviewed by KP (i.e., backward reference search) to identify more relevant studies and manual search was performed in journals most likely related to the topic of this review.

### 3.2 Inclusion and exclusion criteria

The inclusion and exclusion criteria were determined *a priory* and are documented in the study protocol (43). Only studies written in or translated to English were included in this review. Randomised and non-randomised control trials, cohort, case-control, and cross-sectional studies were included in this review. Only studies including at least one group of healthy artistic gymnasts were included. No restriction on age and sex were enforced even though the authors were aware that considerable differences in performance of jumps and landings related to sex might be observed. Only studies including floor exercise without restriction on a specific type of the motor task performed (e.g., drop jump, vertical jump, acrobatic elements) were considered. Furthermore, studies that described kinematics or kinetics during landing without special attention to muscle function or stability in terms of a computation of time to stabilisation or other stabilisation indexes were excluded.

### 3.3 Screening

The results from all searches (i.e., databases and additional sources) were imported into the Covidence software (Veritas Health Innovation, Melbourne, Australia). After removing the duplicates, two reviewers (KP and LB) independently screened all studies by title and abstract using the abovementioned criteria that were inputted into Covidence to ensure consistency between reviewers. Studies meeting all criteria were eligible for full-text review. Conflicts between reviewers were resolved by a third reviewer (MJ) when necessary, as stated in the study protocol (43).

### 3.4 Data extraction

Data from the eligible studies were extracted within Covidence based on the custom data extraction form independently by two reviewers (KP and LB). The custom form was assessed by two reviewers (KP and LB) while extracting data from one eligible study and after final adjustments used for data extraction. Data extraction was focused on the aim and hypotheses of the eligible studies, description of participants, methodological aspects of studies and results related to the aim of this review. Once data extraction was completed, data were transferred into the online MS Excel (Microsoft Corporation, Redmond, WA, USA) spreadsheet and double-checked for clarity (KP). Conflicts between reviewers were addressed and validated by a third reviewer (MJ) when necessary.

### 3.5 Data synthesis

The data synthesis included a summary of the extracted data of the eligible studies and was presented using a combination of descriptive and narrative synthesis. The studies were divided according to the focus of investigating (e.g., research question) and included an overview of the included groups of participants, the motor tasks performed, and the data acquisition and processing details.

## 4 Results

**Figure 1:**
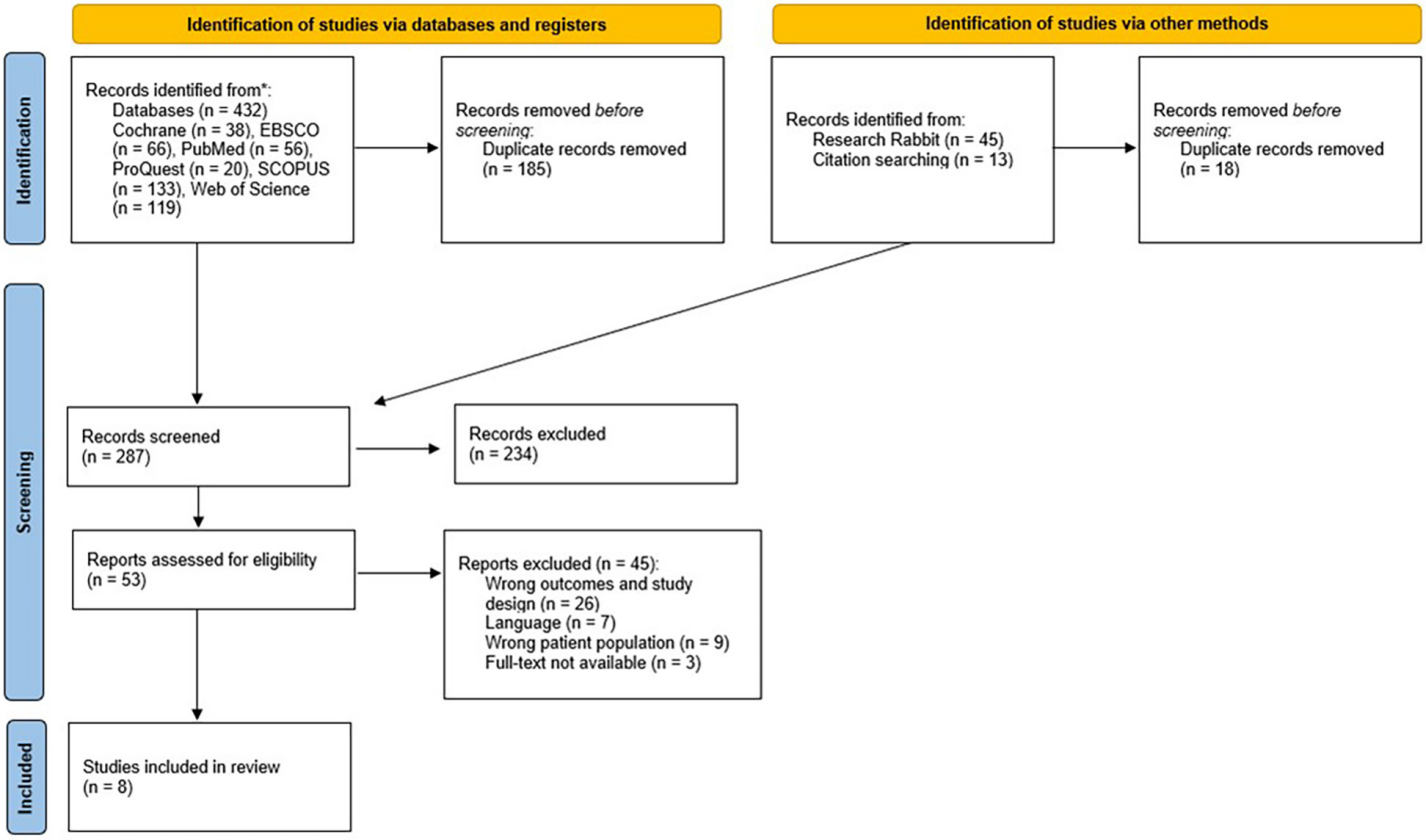
PRISMA flow diagram.

The initial search yielded 432 studies from which 185 duplicates were removed. With including studies from forward and backward search, a total of 53 studies was eligible for a full-text review. Forty-five studies were excluded based on the full-text review after applying inclusion and exclusion criteria stated above. Eight studies were deemed eligible for inclusion in this review (Figure 1, Supplementary Table 2). Six studies focused on muscle function alongside landing kinematics or kinetics, while two studies examined landing stabilization in conjunction with static balance tests. The publication dates of these studies span 20 years, from 2001–2021.

### 4.1 Observed groups

The eligible studies included a total of 212 participants (55% male, 34% female). One study, comprising 24 participants, did not specify the sex distribution. Total sample sizes across studies ranged from 6–104, with individual group sizes ranging from 6–22 participants. Participants were either grouped into a single cohort or divided into as many as six groups. The participants' ages ranged from 8–25 years. Five studies exclusively included adults, two studies examined multiple age groups, and one study focused solely on children. In terms of health status, one study included participants with chronic ankle instability, while the other studies either involved healthy participants or did not specify health status in detail. All studies included at least one group of competitive-level gymnasts; no recreational athletes were involved. Three studies included groups of untrained participants, and one study incorporated swimmers for comparative analysis with gymnasts. Although in one of the studies swimmers were included as a control group, the demands of the sports vary considerably. The differing demands of the sports being compared were reflected in distinct muscular adaptations and landing responses.

### 4.2 Motor tasks and effects

Participants performed jumps and either bipedal or unipedal drop landings in six studies (1, 44–48). One study (45) involved a combination of drop landings and acrobatic elements (tucked forward and backward somersaults), while another study (49) focused solely on backward somersaults. The objectives or effects evaluated across the studies varied. Three studies (45, 48, 49) investigated the impact of the motor task or the landing phase, with one of these also comparing athletes from different sports (swimmers vs. gymnasts) (48). Two studies (1, 44) assessed the effect of varying drop heights, though the heights differed across studies (1.0, 1.5, 2.0 m vs. 0.2, 0.4, 0.6 m). Additionally, the influence of training status was considered in one (50) of these studies and was the primary focus of another. The effects of menarcheal age and a specific exercise program were each assessed in one study (46).

### 4.3 Data acquisition and processing

**Figure 2:**
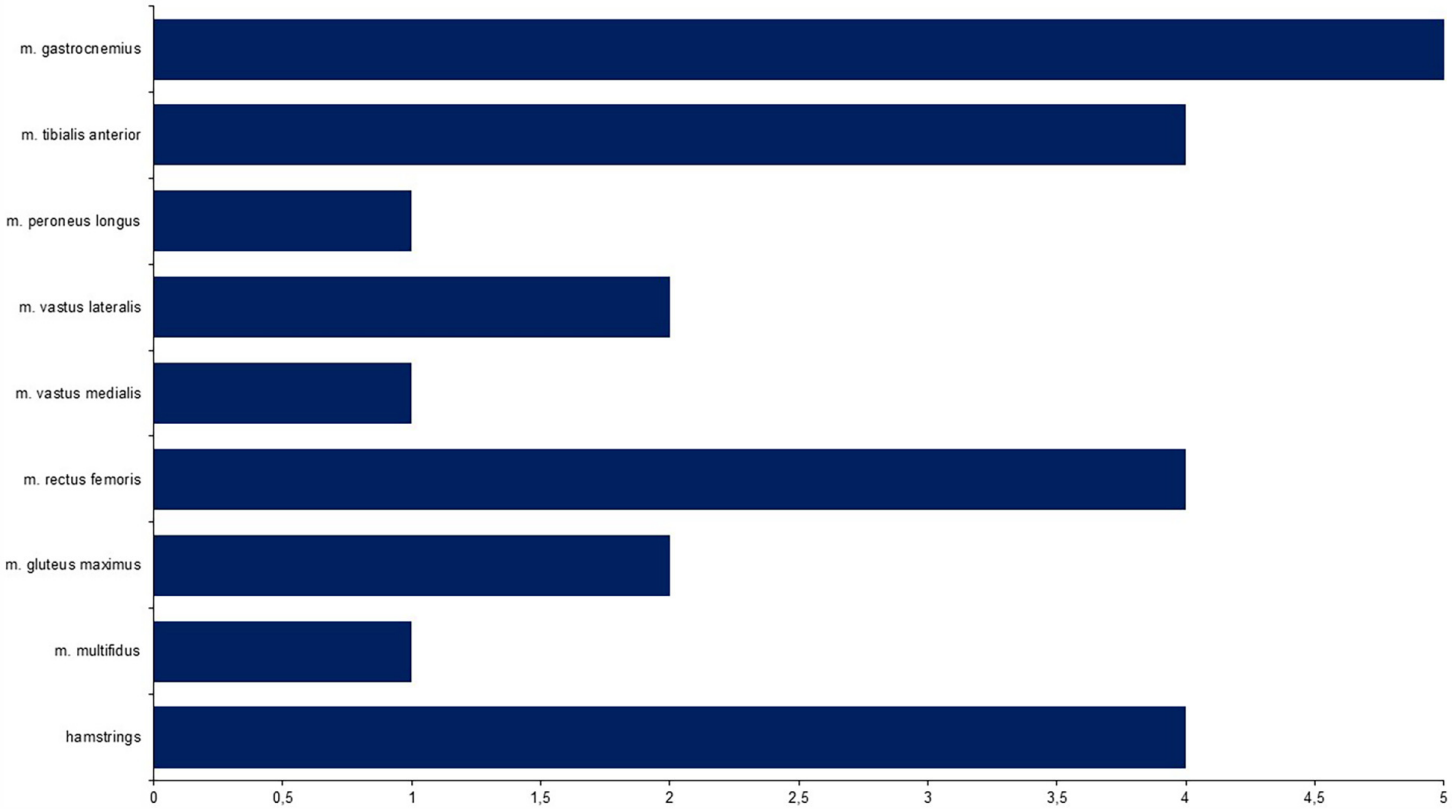
Number of studies analysing specific muscle activity patterns.

Only the data acquisition and processing directly pertinent to the objectives of this review are summarized here, with further details provided in the Supplementary Table 2. The six studies (1, 44–46, 49, 50) that examined muscle function uniformly employed surface electromyography systems, with sampling rates ranging from 1 kHz–2 kHz. A total of ten muscles were investigated across the studies, including the m. gastrocnemius, m. tibialis anterior, m. peroneus longus, m. vastus lateralis, m. vastus medialis, m. rectus femoris, m. biceps femoris, m. semitendinosus, m. gluteus maximus, and m. multifidus (Figure 2).

There was no consensus across studies regarding data processing procedures. All studies applied rectification and filtering processes; however, the sequence and specifics of these procedures varied. Some studies (44, 49, 50) smoothed the signal using root-mean-square (RMS) values, while others used integration or averaging techniques (1, 45, 46). Muscle activity was normalized to a maximal value within each trial, within maximal voluntary contraction, or within the most challenging or the easiest task. The resultant variables ranged from various coactivation indices to average, RMS, or integrated activity during the entire landing, specific landing phases, or within predefined time windows.

The two studies (47, 48) focusing on stabilization also exhibited differences in data acquisition and processing methods. One study (48) used ground reaction force components to assess time to stabilization, while the other (47) analysed centre of pressure movement characteristics (e.g., path length, area) within specified time frames.

## 5 Discussion

The aim of this review was to summarize the methodological procedures used to assess the stability and function of the lower limb and trunk muscles during the landing in gymnasts. Six out of eight eligible studies focused on muscle function during landing, while two studies examined landing stabilization.

### 5.1 Postural stabilisation during landing

Postural control is typically evaluated using postural sway with measures of the distance and amplitude of centre of pressure (COP) displacement (51). A reduction in the path length and area of COP displacement and a reduction in its velocity of movement indicates an improvement in the effectiveness of postural control (52). Measuring postural stability is critical for determining predictors of performance, assessing musculoskeletal injuries of the lower limbs, and determining the impact of physical training and rehabilitation techniques (53).

We identified two studies that focused on the assessment of postural stabilization during landing, with Reis et al. (47) using a pressure plate and Ringhof and Stein (48) using force plates. Reis et al. (47) focused their study on assessing the effect of a neuromuscular reeducation program on postural stabilization during landing after anterior, lateral and medial jumps in healthy gymnasts and gymnasts with chronic ankle instability. A study by Ringhof and Stein (48) focused on monitoring postural stabilization during landing from the single leg jump in female gymnasts and swimmers. Both studies used COP path length and time to stabilisation when assessing stabilisation. In addition, Reis et al. (47) monitored COP area during three intervals—the first two seconds (T1); the second to fourth seconds (T2); and the fourth to sixth seconds (T3).

Although a rigorous examination of the process of postural stabilization during the landing in competitive gymnastics could lead to their improvement and higher scores in competition, no other studies addressing stabilisation during the landing were found. Even so, from the two studies found, we can assume that time to stabilisation might be used for this purpose. On the other hand, we were not able to identify any other types of assessment methods or characteristics (e.g., other indexes, computation based on the signals from inertial sensors or others).

In addition to assessing stabilization of the landing, the study by Ringhof and Stein (48) also looked at other methods of assessing postural stability in competitive gymnasts and swimmers. They used unstable platforms and a controlled fall test. Although they expected gymnasts to perform better than swimmers, gymnasts demonstrated better postural stabilization only during the landing. According to the study by Reis et al. (47), stabilization can be influenced by specialized intervention. Authors focused on the effect of neuromuscular training on stability on the landing in chronic ankle joint instability in female gymnasts. They observed statistically significant improvements in COP displacement and time to stabilisation in the experimental group. The control group that received the same treatment also showed improvement, indicating the possibility of using neuromuscular training as injury prevention and performance improvement.

Taken together, identified studies confirmed that specific training can influence the stabilisation during landing, however, other influencing factors were not found. Studies reporting age (46) or drop landing height (44) affecting lower limb kinematics and muscle function were also found, however, no direct implications to stability indexes were found.

### 5.2 Muscle function of the lower limbs and trunk during landing

Six eligible studies incorporated muscle activity assessment during landing into their data acquisition protocols. All studies evaluated lower limb muscles, with one (50) also including trunk muscles, such as the m. multifidus. A range of techniques was used in data processing, with rectification and filtering (or smoothing via RMS) consistently applied across all studies. Integrated activity, averaged activity, or RMS values were analysed during specific landing phases or within short time intervals (e.g., 20 ms) in the pre-landing and landing periods. Additionally, activation ratios and coactivation indexes were employed in several studies (1, 46, 49) to provide further insights into neuromuscular control mechanisms during landing.

Numerous factors influence muscle activity during landing, including drop height, skill level, age, and task-specific demands. For instance, studies by Arampatzis et al. (44) and Christoforidou et al. (1) investigated the effect of drop height, revealing increased pre-activation and braking-phase muscle activity with greater heights. The former highlighted that gymnasts exhibit earlier muscle activation compared to untrained individuals, suggesting enhanced neuromuscular preparation. Furthermore, gymnasts displayed higher m. gastrocnemius lateralis and lower m. tibialis anterior activity, reflecting optimized joint stabilization strategies. Similarly, Niespodziński et al. (50) found that training adaptations, such as superior muscle coordination and joint stability, were present among gymnasts compared to untrained participants. These variations underscore the influence of neuromuscular control strategies shaped by individual. Among trunk muscles, m. multifidus activity was significantly lower in gymnasts compared to untrained individuals, potentially due to differences in core stabilization strategies (50).

Age-related differences were explored by Niespodziński et al. (50), who found higher muscle activity in younger individuals. Children aged 8–10 years showed higher muscle activity compared to participants aged 12–14 and 18–25 years, which in this study is justified by the fact that muscle activity decreases from childhood through adolescence and into adulthood during specific tasks. Here, the reduction in muscle activity is associated with maturation of the nervous system and therefore improved neuromuscular control. Adult gymnasts modulate their overall body stiffness in response to the landing surface (45), young gymnasts are not yet able to effectively modulate landing strategy like their older peers. Therefore, the scoring system is adapted by individual countries according to their competition categories, taking into account factors such as the use of extra landing mats in younger groups. Kim and Lim (46) reported that post-menarcheal gymnasts demonstrated an increased hamstring-to-quadriceps muscle activity ratio compared to their pre-menarcheal counterparts. However, hamstring recruitment itself was higher in the pre-menarche group, indicating that relative changes in muscle coordination patterns may occur following menarche. Enhanced quadriceps activation during landing preparation may contribute to greater knee loading especially when not counterbalanced by sufficient hamstring co-activation. The co-contraction of the hamstrings and quadriceps remains essential for knee joint stability.

In task-specific analyses, variations in landing kinematics, such as trunk position, also influenced muscle activity. A more horizontal trunk position increased m. gluteus medius activity while reducing biceps femoris activity (45). Similarly, joint stabilization during complex landings was linked to coordinated hamstring-quadriceps activity to counteract excessive knee movement (46).

Distinct patterns of muscle activity emerged during different landing phases. Pre-landing activity, critical for joint stabilization, was pronounced in the m. gastrocnemius medialis (44), while post-landing activity peaked in muscles such as the m. tibialis anterior and m. peroneus longus around 150 ms after ground contact. The hamstrings reached peak activation just before landing, contrasting with the m. vastus lateralis, which was active earlier (80–90 ms pre-landing).

Despite all the abovementioned findings, no consensus on the methodology for muscle activity assessment during landing has emerged. However, given the observed patterns of muscle preactivation and the duration of the braking phase, standard protocols typically involve assessing muscle activity within a 300 ms window before and after initial contact with the ground. Notably, none of the reviewed studies conducted correlation analyses between stabilization indexes and muscle activity or coactivation indexes. Furthermore, no study was identified that examined stabilization indexes during landing in relation to lower limb or trunk muscle strength.

## 6 Limitations

This scoping review is subject to several limitations that warrant consideration. First, discrepancies exist between the registered protocol and this manuscript, primarily concerning the type of landings involved. Specifically, the focus was narrowed to floor exercises of artistic gymnastics. This adjustment was necessitated by the challenges in directly comparing muscle activity during landings from diverse types of jumps, such as ballet/acrobatics jump (as seen in rhythmic gymnastics), bipedal and unipedal drop jumps, and drop landings. The biomechanical differences inherent in these tasks limited the feasibility of drawing meaningful comparisons across varied landing contexts.

Second, a slight reformulation of research question 3 was made during the review process to refine the focus specifically on muscle function during landing rather than more broadly. This modification was intended to align the question more precisely with the review's objectives and prevent potential misinterpretation. While this adjustment enhances clarity, it represents a minor deviation from the initially registered protocol. Research question 4 was also reformulated to ensure clarity, without the deviation of the meaning from the original formulation.

Third, studies that described kinematics or kinetics of landing without addressing stabilization or muscle activity during landing were not considered. Although this decision could be viewed as a limitation, the review intentionally prioritized studies that directly examined stabilization or muscle function during landing. This focus aligns with the author's aim of understanding neuromuscular control rather than solely describing the movement patterns of landings.

Finally, we were unable to address the fourth research question—Which specific lower limb and trunk muscles' strength or activation levels are most frequently linked to landing stability in gymnasts?—as no studies were identified that combined assessments of muscle strength and activity with stabilization metrics. This gap in the literature highlights a significant area for future research to explore the interplay between muscle strength, activity, and stabilization during landing in gymnasts.

## 7 Conclusion

This scoping review mapped methods used to assess the biomechanics of landing in gymnasts, with a focus on muscle function and stability. The findings indicate significant variability in methodological approaches across studies, highlighting the lack of standardization in assessing postural stabilization and muscle activity. Only a limited number of studies have explored postural stabilization during landing, with time to stabilization and COP metrics being the primary measures. Although evidence suggests that neuromuscular training can improve stabilisation during landing, comprehensive insights into other influencing factors, such as age, sex, or landing height, remain limited.

Similarly, the analysis of muscle function revealed diverse protocols for data acquisition and processing, but no consensus on best practices. Gymnasts demonstrate enhanced neuromuscular control compared to untrained individuals, with distinct patterns of muscle activity across landing phases. Factors such as skill level, drop height, and task-specific demands influence muscle activity; however, these effects are inconsistently reported. Importantly, the relationship between muscle activity patterns and stabilization indexes, as well as the role of muscle strength during landing, remains unexplored in the reviewed literature.

Overall, while the reviewed studies provide valuable insights into the biomechanics of landing in gymnasts, future research should prioritize methodological standardization and explore the interplay between muscle function, stability metrics, and achieved score to advance both scientific understanding and practical applications in gymnastics.
